# An Automated Wireless System for Monitoring Concrete Structures Based on Embedded Electrical Resistivity Sensors: Data Transmission and Effects on Concrete Properties

**DOI:** 10.3390/s23218775

**Published:** 2023-10-27

**Authors:** José Roberto Tenório Filho, Jasper Goethals, Reza Aminzadeh, Yawar Abbas, Dulce Elizabeth Valdez Madrid, Veerle Cnudde, Günter Vermeeren, David Plets, Stijn Matthys

**Affiliations:** 1 Magnel-Vandepitte Laboratory, Department of Structural Engineering and Building Materials, Ghent University, 9052 Ghent, Belgiumstijn.matthys@ugent.be (S.M.); 2 IMEC-WAVES, University of Ghent/IMEC Ghent, 9000 Ghent, Belgium; jasper.goethals@ugent.be (J.G.); gunter.vermeeren@ugent.be (G.V.); david.plets@ugent.be (D.P.); 3 Unitron NV, 8970 Poperinge, Belgium; 4 IMEC at Holst Centre, 5656 AE Eindhoven, The Netherlands; 5 PProGRess-UGCT, Department of Geology, Ghent University, 9000 Ghent, Belgium; veerle.cnudde@ugent.be; 6 Department of Earth Sciences, Utrecht University, 3584 CS Utrecht, The Netherlands

**Keywords:** concrete structures, structural monitoring, resistivity, wireless communication

## Abstract

Modern infrastructure heavily relies on robust concrete structures, underscoring the critical need for effective monitoring to ensure their safety and durability. This paper addresses this imperative issue by introducing an innovative automated and wireless system for continuous structural monitoring. By employing embedded electrical resistivity sensors coupled with a wireless-based data transmission mechanism, real-time data collection becomes feasible. We provide a general description of the system’s architecture and its application in a pilot study covering the effects of the devices on concrete properties and data transmission. The dielectric properties of concrete specimens were investigated under natural and accelerated curing/degradation and the results were used in the final design of the antenna device. Furthermore, a pilot test comprising four reinforced concrete columns was used to investigate the range of data transmission from inside to outside of the concrete, the effects of the hardware device on the compressive strength and concrete distribution in the columns, and the data transmission quality in real time under realistic exposure conditions.

## 1. Introduction

Concrete structures form an essential foundation for modern society, serving as the backbone of various infrastructural developments, including buildings, bridges, highways, and dams. As these structures are subjected to numerous environmental and operational factors, their durability and safety become critical concerns. The early detection of structural degradation and performance issues is imperative to prevent catastrophic failures, ensure public safety, and optimize maintenance efforts. In this context, structural health monitoring (SHM) has emerged as an indispensable tool to assess the integrity and performance of concrete structures [1,2,3]. To monitor concrete structures effectively, a range of monitoring systems have been developed and employed in various engineering applications, focusing, among others, on monitoring cracking [4,5,6], corrosion of reinforcing steel [7,8,9], and excessive deflections [10,11].

Another very important aspect in monitoring concrete structures is strength development, especially at early ages. Depending on the concrete type, strength development after 3 days can already amount to 40 to 70% of the 28-day strength, which could enable, for example, a faster removal of formworks or advancements in the construction stage. However, real-time monitoring of concrete strength using non-destructive methods is still a big challenge and a major source of uncertainty. In this context, maturity methods for in situ estimation of concrete strength, based solely on temperature, have been widely used [12,13,14,15] and many commercial solutions have been developed. But these might not always be the most accurate, as the maturity and related compressive strength are also influenced by concrete mixture and the weather (as reported by Yang et al. [12], differences of up to 30% between estimated and real strength could be obtained when using maturity methods for certain types of cement and curing conditions).

In terms of data collection and transmission during the monitoring of concrete structures, traditional wired monitoring systems have been in use for decades and rely on physical cables to connect sensors to data acquisition units. These wired systems often involve significant installation efforts, especially in existing structures, which can be both time-consuming and expensive [16]. Moreover, the presence of cables may introduce potential damage to the structure and hinder the aesthetic aspects. In recent years, wireless monitoring systems have gained considerable attention in the field of structural health monitoring [17,18,19,20]. Unlike their wired counterparts, wireless systems do not require physical cables, offering numerous advantages that significantly enhance monitoring efficiency and overall system performance; among these are flexibility and easy installation, scalability, enhanced data accessibility, and reduced structural impact. However, when working with wireless systems, sufficient attention should be given to in-to-out data transmission and communication.

Wireless communication of antennas within concrete structures poses a unique set of challenges due to the high attenuation and signal loss caused by the dense and absorbent nature of concrete [21]. The propagation of radio waves through concrete is affected by its composition and thickness, resulting in significant path loss. To address these challenges, researchers have explored techniques such as the utilization of innovative antenna designs to enhance the penetration of signals into concrete structures [22]. As wireless communication continues to be crucial for various applications within and beyond concrete structures, ongoing research efforts are vital to optimize signal propagation and coverage in this challenging context. 

Regarding monitoring techniques, the utilization of electrical resistivity (ER) measurements within concrete emerged in several studies as an indicative measure of moisture content and durability [23,24]. Furthermore, investigations have explored associations between ER measurement and the compressive strength of concrete as it matures. This measurement entails assessing ER either at the surface or through embedded electrodes within the concrete. Key factors influencing ER include (1) concrete pore structure, (2) moisture levels, and (3) the presence of ions, such as chloride, within the concrete matrix. 

ER measurement with embedded sensors in fresh concrete serves as a gauge for the efficiency of the curing process and serves as an indicator for the long-term durability of concrete. However, existing ER-based sensors in the literature or commercially available ones tend to be costly or limited to single-point measurements in the concrete cover depth [25]. To ensure reliable and real-time assessment of concrete hydration and durability, an embedded ER sensor is essential to map resistivity profiles across the concrete cover depth. 

Considering all the points mentioned above regarding the needs and challenges for new and more efficient monitoring devices and systems for concrete structure, in this study, we describe a novel, automated, and wireless system for monitoring concrete structures based on embedded electrical resistivity sensors. This solution could be applied to monitor strength development in real time at early ages, and durability aspects such as the progress of carbonation at later ages, overcoming the drawbacks of traditional solutions that in most cases depend on wired connections for data collection and/or transmission (or even with wireless data transmission, but this is based on Bluetooth Low Energy (BLE), limiting the wireless readout range to only “a couple of meters”), and rely solely on temperature data for maturity and strength prediction. This paper covers a general description of the system’s architecture and its application in a pilot study investigating the effects of the devices on concrete properties and data transmission. 

## 2. Experimental Section

The experimental setup consisted of laboratory-scale testing for the determination of dielectric properties of concrete mixtures, which were afterwards used for the construction of a pilot case composed of reinforced concrete columns. The reinforced concrete columns were used to test (1) the effects of the embedded devices on the strength and microstructure of the concrete, (2) the data collection and transmission in the presence of steel reinforcement, and (3) the antenna range under more realistic conditions. In the following subsections, details are given for the concrete mixtures used in the study, as well as the production and testing of specimens. Before detailing the test methods, an overview of the system, from now on named “the COMMODORE solution”, is presented.

### 2.1. Description of the “COMMODORE” Solution

**Figure 1:**
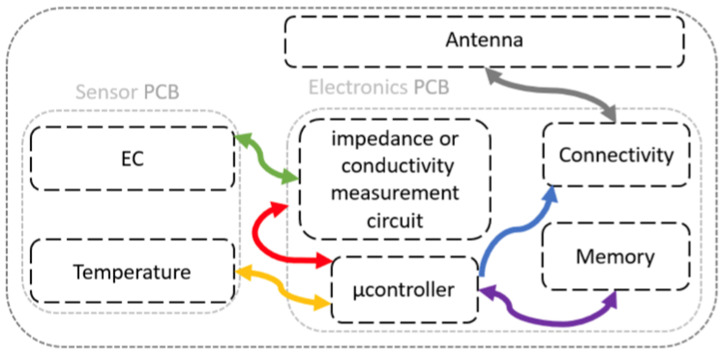
High-level architecture of the hardware electronics and interconnections with the sensor board and antenna.

Figure 1 presents an overview of the high-level architecture of the hardware electronics. This hardware configuration comprises three distinct printed circuit boards (PCBs): (1) the sensor board; (2) the electronics board operating as the central processing unit and managing key functionalities; and (3) the antenna PCB. 

#### 2.1.1. Sensor Design

**Figure 2:**
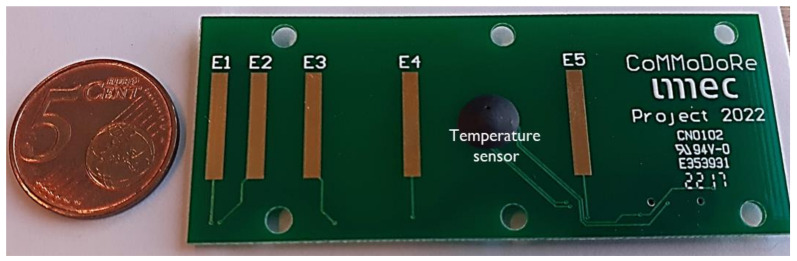
Snapshot of the concrete resistivity sensor.

The concrete resistivity sensor is shown in Figure 2. The sensor is composed of five arrays of electrodes, measuring concrete resistivity between different electrode pairs. The approach is to design an array of electrodes, with the two-electrode measurement principle, where the spacing between arrays of electrodes closer to the concrete surface needs to be smaller to obtain a higher resolution of measurements close to the concrete surface. With such an electrode, it is possible to monitor concrete resistivity at four different concrete cover depths. 

The sensor was constructed with hard-plated gold electrodes on a PCB substrate. The fabrication technique is in line with standard PCB board manufacturing. Hence, there is potential for cost reduction with mass fabrication. The spacing between the electrode pairs over the concrete cover depth and their respective cell constants are given in Table 1. These distances were chosen to collect higher spatial resolution resistivity measurements close to the concrete surface, where the change in resistivity values is higher compared to the innermost depth (50 mm) of the concrete cover.

The sensor operates at an excitation amplitude of 17 mV and frequency of 3 kHz. At this frequency, the phase angle is 0 degree, and therefore the impedance measurement of the ER can be used as a measure of the resistivity of the concrete. The sensor along with the readout and wireless communication module are enclosed in a compact housing. Initially, the value of 17 mV excitation voltage was chosen to design and calibrate the sensors. This value was carefully chosen for this electrode design so that the resulting current in concrete stayed within the instrumentation limits. The range of current measurement is from 2 nA to 90 µA. Instrumentation details of the measuring electronics are provided in [26]. The designed electronics used in the final solution, which were applied in the concrete columns described in the study, provide a sine wave with an amplitude of 800 mV.

#### 2.1.2. Hardware Electronics 

All the hardware components are protected from the surrounding harsh environment (inside concrete) by a 3D-printed housing. The housing material is polyamide 11 (PA11) and is printed using the SAF (Selective Absorption Fusion) 3D-printing technology. In addition, three lithium AA batteries are placed inside the housing to provide the required power for operation of all the units. 

The electronics PCB acts as the hub for impedance measurements through the designed sensor board. This function is orchestrated by a Cortex-Arm processor, the central processing unit of this module. By seamlessly integrating with the connectivity module (Quectel BG95-M6), wireless communication capabilities are enabled. This module is based on Low-Power Wide-Area (LPWA) technology, ensuring energy-efficient, long-range wireless connectivity. The PCB includes a SIM card slot that connects to the connectivity module. This combination of the SIM card and connectivity module harnesses the potential of Narrowband Internet of Things (NB-IoT) and LTE Machine-Type (LTE-M) communications, as standardized by 3GPP. NB-IoT emphasizes extended battery life, cost-effectiveness, and high connection density. In contrast, LTE-M offers greater speed, optimizing communication duration.

During communication sessions, the Quectel module assesses both NB-IoT and LTE-M networks, automatically selecting the one with superior connectivity. In scenarios where network access is unavailable, measurements are temporarily stored for subsequent transmission. Following each data exchange, the information is securely stored within the Unitron cloud (uCloud), allowing for comprehensive data analysis.

#### 2.1.3. Antenna Design

As highlighted earlier, the existing literature presents several approaches for transmitting data from nodes to a database. These methods include wired solutions [27], short-range wireless options like Bluetooth Low Energy (BLE) [28], and the establishment of local wireless sensor networks (WSNs). To address the limitations of wired and short-range wireless methods, which require on-site personnel for data collection, we propose a long-range wireless solution. The long-range solution has the added benefit of eliminating the need for a local gateway, as seen in WSN approaches. This study introduces a practical in-concrete embedded Narrow-Band Internet of Things (NB-IoT) [29] solution that operates within the LTE network infrastructure, thus avoiding the need for additional on-site installations. To enable the NB-IoT connection from the embedded antenna to the LTE base station, we investigated the electromagnetic properties of concrete that influence the antenna’s performance. The importance of an optimized communication link is to optimize the battery lifetime of the device. Despite only sending a maximum of 32 kB per day, communication requires a significant part of finite energy. To make sure that the monitoring continues for a useful length of time, this link must be optimized. Prior research on in-concrete embedded antennas primarily consists of theoretical explorations [30,31,32] or less mature solutions [33,34,35]. The goal in this paper is to optimize a communication link by designing an in-concrete embedded antenna that is (1) robust against the changes in the concrete dielectric parameter in time, and (2) achieves a range of 100 m using the NB-IoT technology on the LTE band B20 (800 MHz). This way, we achieve a robust communication link from the node inside the concrete structure to the base station located at the cell tower.

#### 2.1.4. Cloud Solution

As mentioned in the previous section the captured data are transmitted to Unitron cloud (uCloud). Data being sent via either NB-IoT or LTE-M are transferred to the uCloud via the MQTT-SN (Message Queuing Telemetry Transport for Sensor Networks) messaging protocol. The selection of MQTT-SN stems from its adeptness in catering to the intricacies of IoT communications, manifesting in robustness, reliability, data integrity, and the perpetuation of sensor longevity via enhanced battery utilization. 

Within the framework of MQTT-SN, the devices, referred to as clients, dispatch messages to a central broker. This broker, in turn, receives these messages and disseminates them to designated subscribers. The classification of each message is contingent on its associated topic—an addressing mechanism assigned to each published message. By subscribing to one or more specific topics, the recipients gain access to messages allocated to those topics.

Upon arrival at uCloud, the transmitted measurements are meticulously stored and systematically organized within a dedicated database. To interact with this database, a web application serves as the interface. Furthermore, accessibility to the database extends to authorized personnel, who can engage in the processing and analysis of the archived measurements. This analytical exploration lends itself to the refinement and visualization of the stored data, enabling a comprehensive understanding of the information at hand.

### 2.2. Concrete Mix Design for the Laboratory Tests (Dielectric Properties of Concrete) and Pilot Test 

Two concrete mixtures were used in this study, chosen to be representative of common applications for structural concrete. The mixtures will be here defined as M1 and M2. Details of the mix designs are presented in Table 2.

### 2.3. Measurement Setup for Dielectric Parameters

For each distinct concrete type used in the columns, specimens in the form of cubes with side length of 150 mm were cast and exposed to two different conditions: (1) cubes were molded and placed in a room with controlled atmosphere (temperature of 20 ± 2 °C and relative humidity of 60 ± 5%), demolded after 24 h, and then stored again in the same room during the whole monitoring period of three months; (2) cubes were molded and placed under curing conditions for 28 days (also being demolded after 24 h) and then transported to a carbonation chamber (temperature of 20 ± 2 °C, relative humidity of 60 ± 5% and CO_2_ concentration of 1%) where they remained during the whole monitoring period. Cubes were used to facilitate the measurements and to be able to assess the connectivity given the permittivity of the used concrete. This was to make sure that we achieved the desired range given the design and the used RF technology. The measurement protocol entailed selecting two smooth surfaces on each cube to ensure consistent and stable readings. Three distinct locations were chosen on each surface to account for the inherent heterogeneity of concrete composition. These designated locations were consistently measured at each time point to ensure accurate tracking of permittivity evolution. This procedure was iterated for every concrete type and aging condition under investigation. 

**Figure 3:**
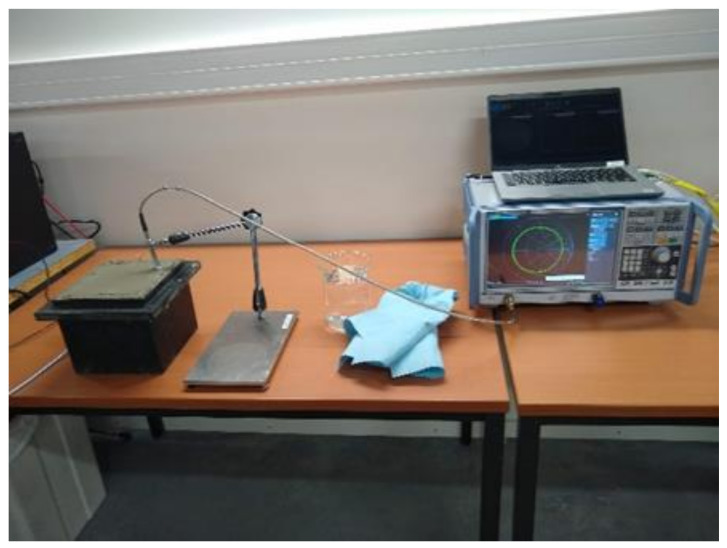
The dielectric measurement setup with the Speag DAK connected to the R&S VNA. In the picture, the concrete cube is still in its mold.

Open-ended coaxial measurements were performed using the Dielectric Assessment Kit (DAK) of SPEAG (Zürich, Switzerland) in conjunction with the Rohde & Schwarz ZNB20 vector network analyzer (VNA), see Figure 3. To calibrate the DAK, the open–short–load method was employed, employing 1-propanol as the reference substance. The permittivity measurements were conducted within the frequency range of 200 MHz to 3 GHz, employing a resolution of 2 MHz. 

### 2.4. Description of the Pilot Test

**Figure 4:**
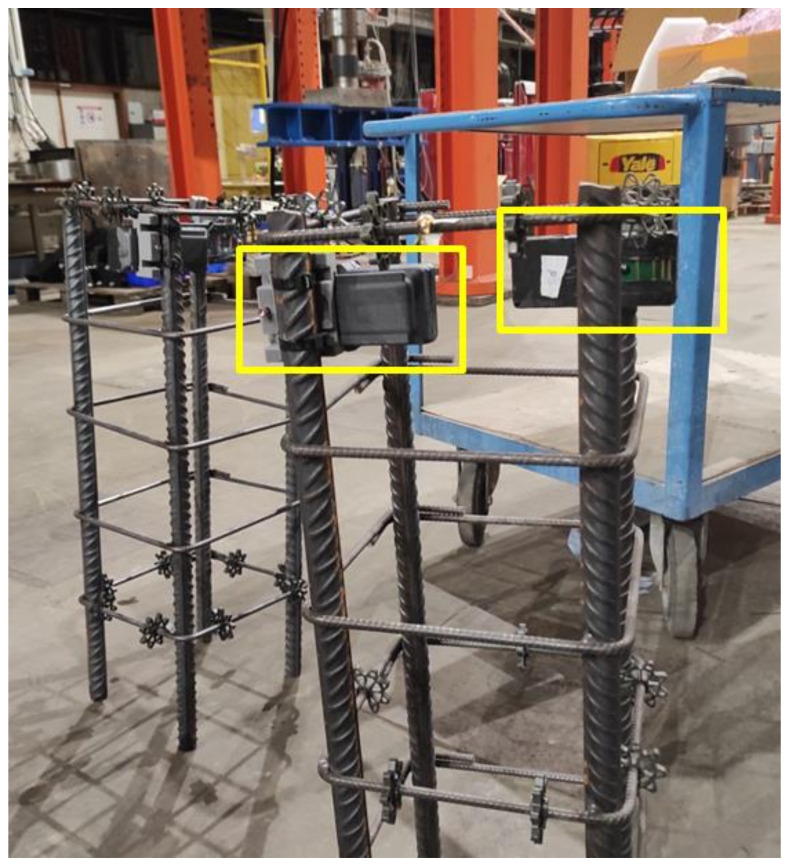
Reinforcement cages of the concrete columns before placing in the formwork. The hardware devices are highlighted.

**Figure 5:**
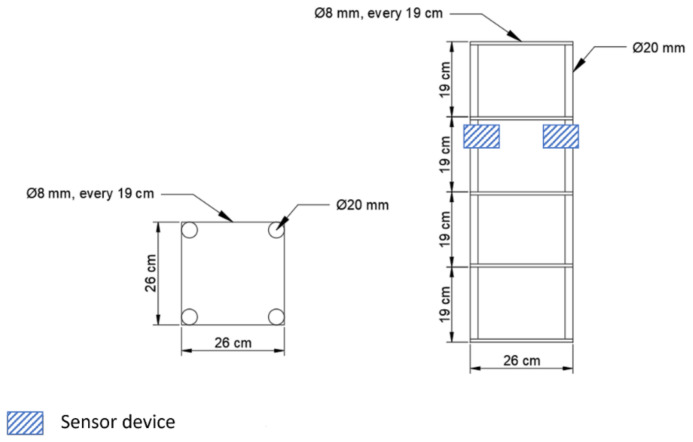
Details of the reinforcement cage.

For testing with the embedded wireless sensors, four reinforced concrete columns were built. The columns had dimensions of 320 mm × 320 mm × 820 mm and a concrete covering of 30 mm. Three of them were fabricated with concrete M1 and the fourth column with concrete M2. In each column, two devices were embedded (Figure 4), one for the collection of electrical resistivity and the second to test the range of the antenna for data transmission. The reinforcement of the columns was composed of a reinforcement cage with four vertical rebars with a diameter of 20 mm and horizontal rebars with a diameter of 8 mm, placed every 19 cm (see Figure 5). The sensors were placed facing opposing surfaces of the columns, right below the second horizontal rebar (from the top of the column). After casting, the columns were moved to a room with an average temperature of 20 ± 2 °C and relative humidity of 60 ± 5%. They were demolded after 24 h and then transported to a testing area outdoors where they were subjected to uncontrolled and realistic environmental conditions. During the testing time, both environmental temperature and relative humidity were monitored using an external device placed near the columns.

### 2.5. Testing the Effects of the Sensor Device on the Concrete

After the data collection, the concrete columns produced with concrete M1 were used to further investigate the effects of the sensor device on the concrete properties. Two of them were used to verify the effects of the embedded devices on the compressive strength of the concrete and the third one to verify the influence of the device on the distribution of concrete around the sensor.

**Figure 6:**
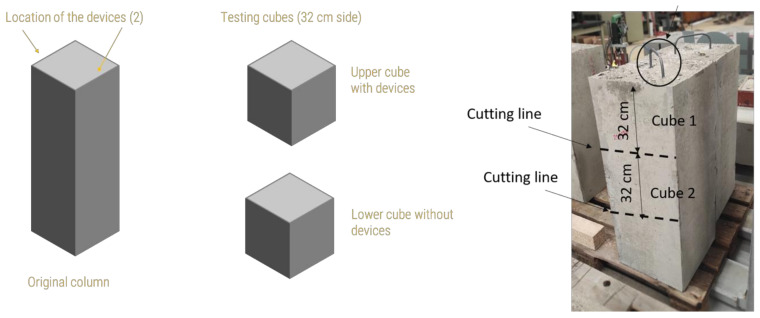
Schematic of the cutting of the columns to obtain the testing cubes. The circle-Transportation hook.

**Figure 7:**
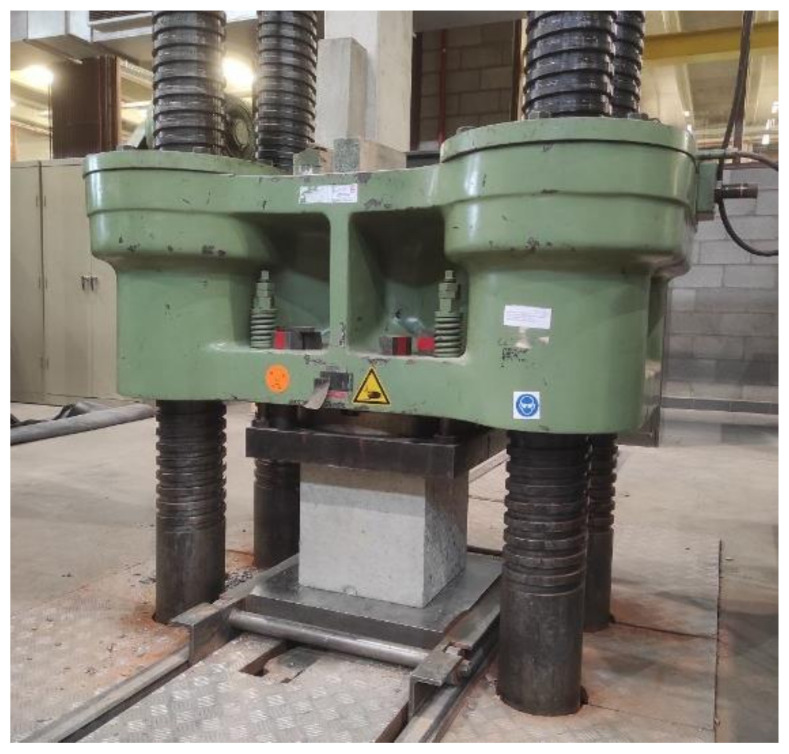
Testing machine used for assessing the compressive strength of the cubes.

To test the compressive strength, two cubes with dimensions 320 mm × 320 mm × 320 mm were cut from two of the reinforced concrete columns (Figure 6). The cube cut from the upper part of the column contained the two housing devices embedded in the column, while the cube cut from the middle part of the column was fully composed of reinforcement and concrete, without any devices. The cubes were tested 99 days after the casting of the columns. A Wolpert testing machine with a load capacity of 10,000 KN was used, as shown in Figure 7.

**Figure 8:**
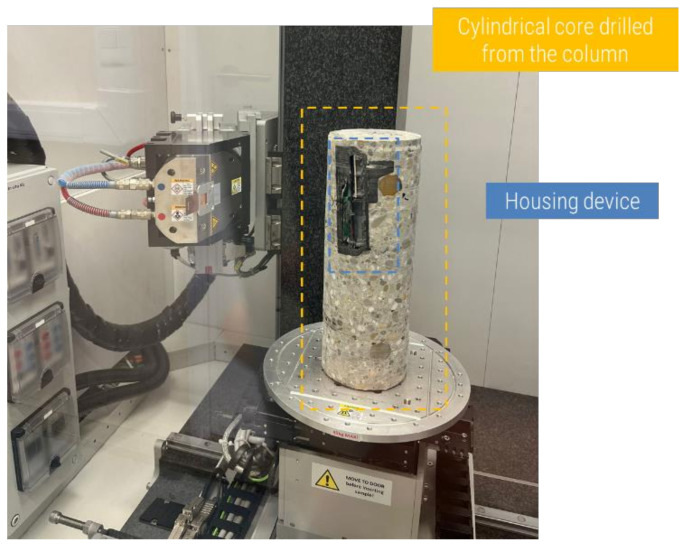
Setup for the scanning of the cylindrical core drilled from the column with UGCT’s CoreTOM scanner.

To investigate possible influences of the presence of the housing device on the distribution of concrete inside the column and possible increase in the air content/voids near the devices, a non-destructive and three-dimensional technique such as micro-computed tomography (micro-CT) was required [36]. A cylindrical core with a diameter of 115 mm and height of 320 mm was drilled from the upper part of the third column produced with concrete M1. The cylinder was composed of two regions: one affected by the presence of the housing device, and another without the device. The cylinder was then scanned using the TESCAN CoreTOM system equipped with a 180 kV X-ray tube and a large 2856 × 2856 pixels flat panel detector (see Figure 8). A stack of 4 scans of the core were taken to cover the entire volume, reaching a resolution of 90 μm. A 1.5 mm Cu filter, 180 kV, and 90 W were required to reduce the noise given by the steel reinforcement of the concrete. A total of 2142 projections with an exposure of 650 ms were captured per scan. The reconstruction of the volume was achieved using the Panthera 1.4.4 software by TESCAN XRE, and the image analysis was carried out using Avizo 2020.3.

The quantification of the air voids was carried out by separating the reconstructed volume into two regions: region 1, containing the housing device; and region 2, formed by the bulk of the reinforced concrete core. A total of 1520 slices were used for the analysis of region 1, and 1204 for region 2. Firstly, a segmentation of the voids was performed to isolate them from the rest of the core. Afterwards, each void was assigned geometric properties to quantify the total volume per region. Lastly, a distribution of the different sizes of air voids was obtained. 

**Figure 9:**
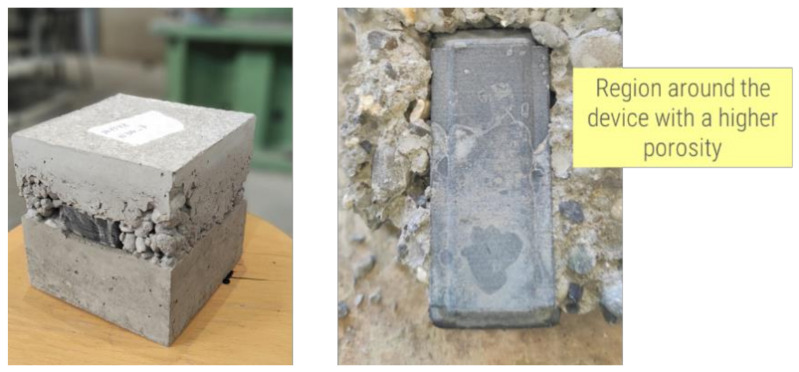
Poor distribution and compaction of concrete around the hardware device.

This investigation was justified based on preliminary tests that showed a significant influence of the hardware on the distribution of concrete and air voids in the areas surrounding the device when it was embedded in a concrete cube with side length of 150 mm (Figure 9).

### 2.6. Verifying the Antenna Range 

To verify the quality and robustness of the wireless connection of the presented solution, we monitored the Received Signal Strength Indicator (RSSI) metric of the uplink channel, i.e., of the packages from the sensor nodes to the cell tower, over the course of a month while the nodes were embedded on the rebar in the concrete columns. During this time, the concrete was poured, the concrete dried, and the structure was demolded and moved. This highly variable environment for the node displays the robustness of the communication link. The chip for the communication used is Quectel BG95-M6, using the Proximus network in Ghent, Belgium. 

## 3. Results and Discussion

### 3.1. The Dielectric Properties of Concrete 

Table 3 lists the measured relative permittivity ($\epsilon_{r}$) and conductivity (σ) for concrete M1. After the first 24 h, the relative permittivity drops from 30 to 6. The values are constant after the first day until long after. We concluded that the permittivity of concrete does not change during its lifetime after it has dried out. The values of permittivity can be reasoned as follows. During the first day, the free water will bind to the cement or evaporate, diminishing its contribution to the effective permittivity of the concrete. The permittivity of water is significantly higher than the other ingredients (water has a relative permittivity of 80 at 800 MHz, and the other ingredients range from 4 to 10). The loss of free water explains the significant drop in permittivity after the first day. This reasoning also explains why it remains constant over time. There is no more water to evaporate or to bind to the concrete. 

The observation that the parameters of concrete are constant over time has the consequence that the communication link will not be influenced by the concrete life cycle, by which we mean that the variation in the communication link will not originate from the variation in the concrete parameters, as these are constant. This will ease the robustness requirement during the antenna design. The dielectric parameters will have a large impact due to the dielectric loading of the environment on the antenna. These being constant over time facilitates the design, which is used during the evaluation of Section 3.2. In Table 3, the parameters of the concrete M1 at distinct time intervals are given. 

### 3.2. The Antenna Range 

**Figure 10:**
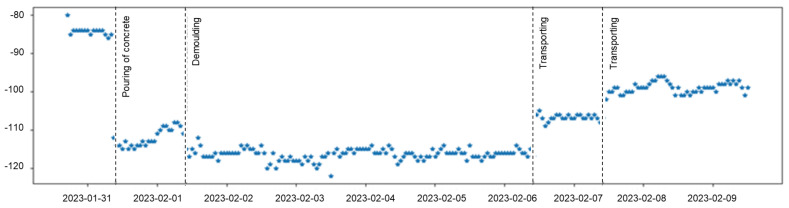
The RSSI values as a function of time. It is clear from the results that the communication link was robust against the many changes in its environment.

The designed antenna will be discussed in detail in a follow-up paper. Figure 10 shows the RSSI values as a function of time. These are the data of only one of the columns with concrete M1, since, during the test, the device placed in the second column faced battery issues. The node was evaluated over a week, until the batteries ran out. On the first day, 31 January 2023, the node was mounted to the rebar. At 10 am, the concrete was poured, resulting in a significant drop in the RSSI value (−30 dB). After a day, the concrete columns were demolded, resulting in a variation (±5 dB) in the RSSI due to the high variability in its environment during demolding. After the demolding, the columns were left to rest inside the laboratory under controlled atmosphere (temperature of 20 ± 2 °C and relative humidity of 60 ± 5%), resulting in a flat RSSI behavior. On the seventh day, the columns were placed outside of the laboratory, exposed to realistic environmental conditions resulting in an uptick of the RSSI (+10 dB). One day after moving the columns outside there was an increase in the RSSI value to –100 dB (+15 dB compared to its inside location). This increase in RSSI is due to the blocking nature of the laboratory (e.g., for a building with reinforced concrete). This difference in reception is well known and characterized in the mobile community (e.g., the 3GPP 3D-UMa model [37]). 

**Figure 11:**
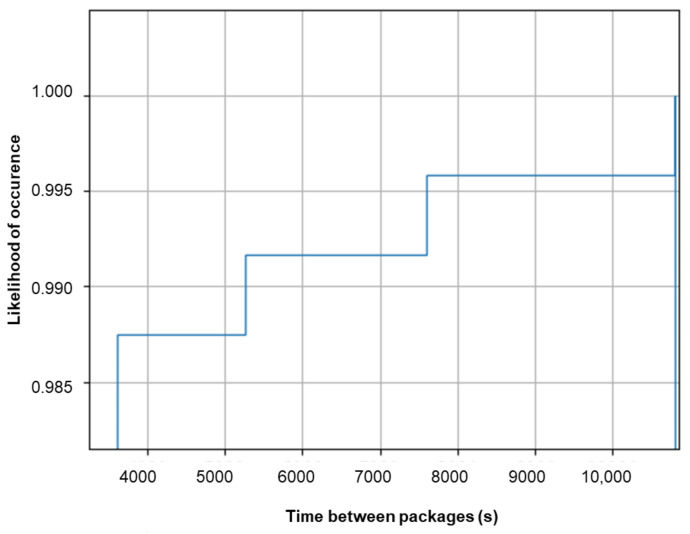
The cumulative distribution of the time between packages as a measure of communication link robustness. Every hour (3600 s), the node tried to send a package. The cumulative histogram shows that more than 98% of the packages were received within time.

The robustness of the communication link is measured as follows. The node tries to send a package every hour. When a package is not received, the node will try to resend the package. Due to the time it takes to resend the package, the time between each package will determine the number of retransmissions (ignoring the drift in the RTS clock of the device). Figure 11 gives the cumulative histogram of the time intervals of the received packages. It shows that more than 98.5% of the packages were received within a second. This is a manifestation of a robust communication link.

**Figure 12:**
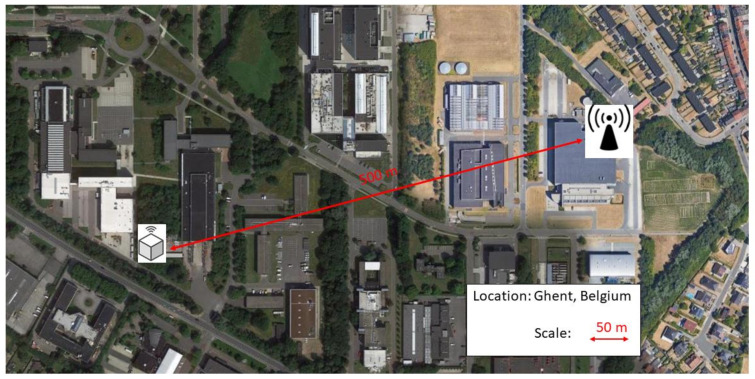
The place of the columns with the nodes and the connected cell tower. A range of 500 m has been achieved.

Figure 12 shows the cell tower with the most retrieved packages. This tower was located 500 m from the transmitting device. Therefore, we conclude that a range of at least 500 m is achieved from the nodes embedded in the concrete to the LTE cell tower, surpassing the predetermined goal of 100 m. We achieved a long-range communication link from in-to-out concrete to an LTE cell tower. 

### 3.3. The Data Transmission to the Cloud

**Figure 13:**
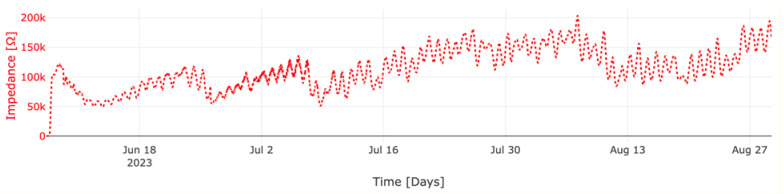
Measured impedance of column M1 between 7 June and 29 August.

**Figure 14:**
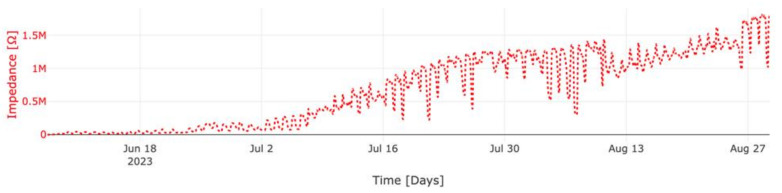
Measured impedance of column M2 between 7 June and 29 August.

Figure 13 and Figure 14 illustrate the measured impedance values of concrete columns M1 and M2, respectively. The provided data for both mixtures correspond exclusively to the electrode pair E4–E5, chosen for its depth within the concrete, rendering the measured data less susceptible to factors such as the water evaporation rate on the surface of the concrete columns.

In the case of mixture M1, an initial impedance of 372 Ohms was observed upon concrete pouring. Over a span of 30 h, this value gradually escalated to 639 Ohms. Subsequently, within one day, a peak value of 120 kOhms manifested—a peak intricately linked to sudden shifts in the internal concrete temperature following cement hydration. These temperature fluctuations saw a rise from 21.7 °C to 39.9 °C. A few hours later, the temperature decreased to 24.6 °C and consequently so did the impedance. Following this thermal upheaval, there was a swift decline in temperature to 24.6 °C, mirrored by a corresponding dip in impedance. Nonetheless, the impedance continued increasing due to the ongoing concrete-hardening process. Notably, the undulations in the impedance curve concurred with the oscillations in temperature. After the passage of 28 days, M1 exhibited an impedance of 126 kOhms.

As for column M2, the initial impedance measured during concrete pouring stood at 570 Ohms. In a manner akin to M1, a temperature surge from 23 °C to 31.1 °C prompted a corresponding 2853 Ohms rise in impedance. Despite subsequent temperature diminution to 25.7 °C, the measured impedance reached 17 kOhms 46 h after the mixture was poured. Evidently, the fluctuations in the resistivity curve mirrored the temperature variations with remarkable fidelity. Upon the culmination of 28 days, the impedance reached 221 kOhms. Comparatively, M2 displayed a more rapid rate of impedance increase than M1. Impressively, after a span of 83 days, M2′s impedance surpassed that of M1 by a factor of 6, which can be linked to the different dynamics of the cement hydration reaction given that M2 was produced with a blended cement type, in which a secondary chemical reaction (the pozzolanic reaction) is expected to take place at later ages, contributing to a densification of the matrix and further strength development. 

After a duration of 83 days, both devices remained operational without any reported issues for the batteries or in data transmission. This serves as a testament to the robustness and dependability of the wireless communication system in use, regardless of the presence of steel reinforcement and cement types. In addition, after 83 days, the batteries were still at their highest capacity, meaning that the current consumption of the nodes in the long term has been stable.

### 3.4. The Effects of the Sensor Device on the Strength and Microstructure of the Concrete Columns

In terms of compressive strength, the cubes extracted from the concrete column in the region without the devices presented an average value of 49 MPa at the age of 99 days. In contrast, the average strength of the cubes containing the two devices was 43 MPa. This represents a reduction of 12% in the compressive strength value due to the presence of the device. However, regarding ordinary applications, this reduction could be considered acceptable. In addition, the configuration of the columns with the devices as presented here also amplifies the negative effects of the devices on the strength, given that in a cross-sectional area of 320 mm × 320 mm (referring to the tested cube), the devices occupy together an area of 12,480 mm^2^ (12% of the total cross section).

**Figure 15:**
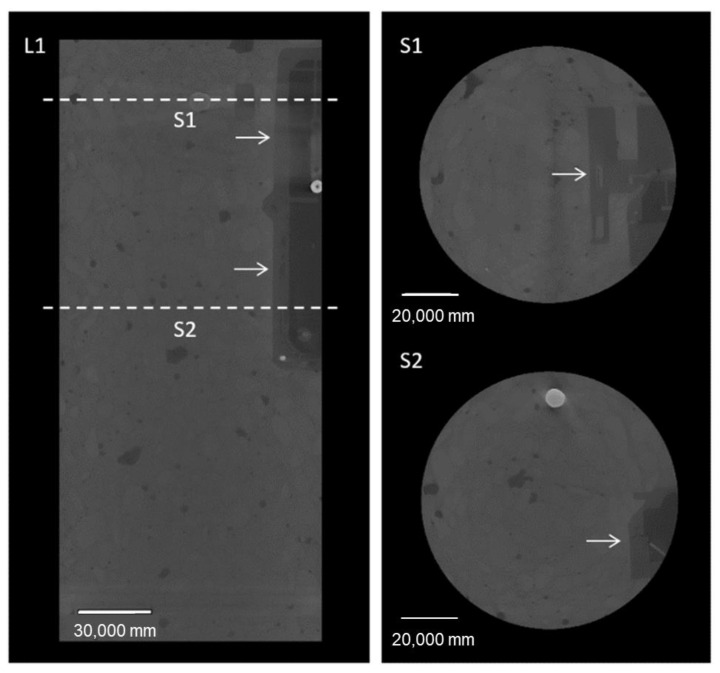
X-ray CT reconstructed cross sections of the concrete core (UGCT CoreTOM, 180 kV, 90 W, exp. 650 ms, 90 µm voxel size). L1—longitudinal section displaying the location of the housing device. S1: top section; S2: bottom section of the device. The arrows show the location of the housing device. Units are displayed in micrometers.

As for the distribution/content of air/voids, it is possible to observe that there is no change in the concrete matrix nor accumulation of air voids in the surrounding areas of the housing device. In Figure 15, one longitudinal (L1) and two perpendicular cross sections (S1, S2) show the housing device embedded in the concrete core. 

**Figure 16:**
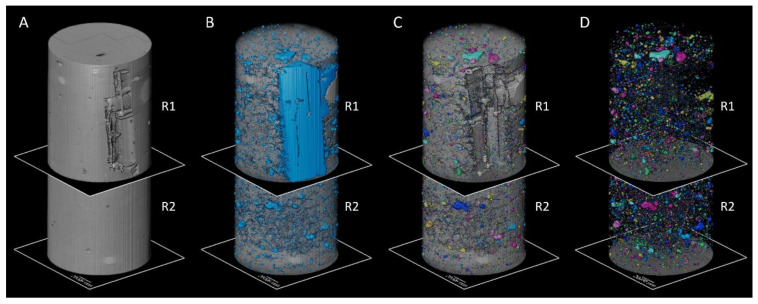
Three-dimensional volumes of the concrete core divided into regions: (**A**) original volume; (**B**) segmented air voids and housing device; (**C**) filtered air voids; and (**D**) isolated air voids. UGCT CoreTOM (180 kV, 90 W, exp. 650 ms, 90 µm voxel size).

The total volume of air voids was quantified to compare both regions. The image analysis workflow (see Figure 16) using Avizo 2020.3 consisted of four main steps. First, the core was subdivided into region 1 (with the housing device) and region 2 (with no device) as shown in Figure 15. Then, the air voids, housing device and reinforcement were segmented, and then assigned geometric attributes. The housing device and reinforcement were filtered out and the total air content was calculated per region.

In Table 4, the total volume of the concrete core and the total amount of isolated air voids per region are shown. The volume of the R1 concrete core was calculated by mathematically subtracting the volume of the housing device. R1 shows an overall higher volume of air voids in comparison to R2. Nevertheless, R2 exhibited a higher density of air voids when calculated relative to the total studied volume in each region.

**Figure 17:**
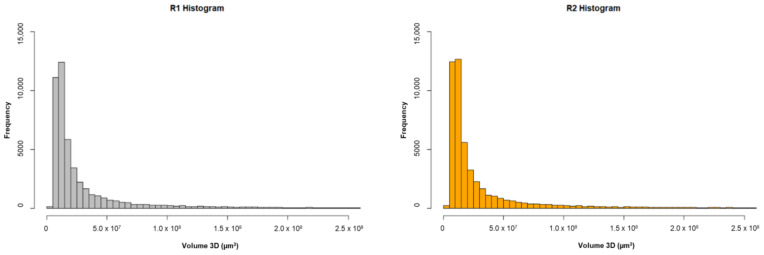
Size distribution of the individual air voids by volume.

Finally, a histogram per region is displayed on Figure 17, showing a remarkably similar size distribution of the air voids by volume. The main difference relies on the slightly higher concentration of particles under 1.0 × 10^7^ µm^3^ on R2 and, therefore, it is not considered that a significant difference exists between both regions due to the presence of the housing device. 

## 4. Concluding Remarks

In this paper, we investigate the feasibility and working mechanisms of a novel solution for monitoring concrete structures based on a wireless electrical resistivity sensor. As an alternative to current commercial solutions, this system has the added features of large-range wireless data transmission and simultaneous recording of electrical resistivity on different depths of the concrete cover. The research covered the determination of the dielectric properties of concrete under semi-realistic curing conditions as well as accelerated degradation conditions, the data transmission and antenna range in the wireless communication of the sensor device embedded in real structures, and the effects of the hardware on properties of the concrete such as compressive strength and distribution of concrete/presence of air voids in the proximity of the devices. In summary, the following conclusions could be drawn from the study:

- The permittivity of concrete does not change during its lifetime after it has dried out, not even under accelerated degradation conditions (accelerated carbonation). Thus, the communication link will not be influenced by the concrete life cycle, which will reduce the robustness requirement during the antenna design stage.
- There was no interference of the reinforcement steel bars on the in-to-out transmission of concrete antenna communication and a long-range communication link from in-to-out concrete to an LTE cell tower was achieved. Thus, wireless communication will not limit the use of this system for any concrete application.
- The sensors and whole hardware device performed in a stable and reliable way during data collection and transmission in the pilot study for the two cement types used under realistic conditions, which means that the system could be used for many applications within the concrete industry (different cement types and different environmental conditions).
- The presence of the hardware device did not promote a poor distribution of the concrete in the areas close to the sensor, and no increased air void or poor adherence between the concrete and the device were found. This proves that the use of the system will not significantly interfere with the properties of the concrete structure.

## Figures and Tables

**Table 1:** Spacing between different electrode pairs of the sensor.

No.	Electrode Pair	Spacing (mm)	Cell Constant (cm^−1^)
1	E1–E2	5	1.08
2	E2–E3	7	1.203
3	E3–E4	15	1.418
4	E4–E5	19	1.591

**Table 2:** Mix design of the concrete mixtures M1 and M2.

Materials [kg/m^3^]	Concrete M1	Concrete M2
Cement type CEM I 52.5 N (Holcim, Belgium)	300	0
Cement type CEM III A 42.5 N LA (Holcim, Belgium)	0	300
River sand 0/4 mm	665	665
Gravel 2/8 mm	575	575
Gravel 8/16 mm	675	675
Water	170	170

**Table 3:** The measured relative permittivity (ϵ_r_) and conductivity (σ) at 800 MHz.

First Day of Pouring		Day after		Three Months after Pouring		Accelerated	
$\epsilon_{r}$ [−]	σ [S/m]	$\epsilon_{r}$ [−]	σ [S/m]	$\epsilon_{r}$ [−]	σ [S/m]	$\epsilon_{r}$ [−]	σ [S/m]
30	1.75	6	0.35	5	0.075	5	0.075

**Table 4:** Quantification of air voids volume per region based on the X-ray CT data.

Region	Number of Slices	Core Volume per Region (µm^3^)	Total Volume of Air Voids per Region (µm^3^)	Air Voids/Volume of Region (µm^3^)
R1	1520	1.42 × 10^21^	1.77 × 10^13^	1.25 × 10^−8^
R2	1204	1.12 × 10^21^	1.60 × 10^13^	1.42 × 10^−8^

## Data Availability

The data presented in this study are available on request from the corresponding author. The data are not publicly available due to project confidentiality.
